# The Juggernaut of Modernity Collapses. The Crisis of Social Planification in the Post COVID-19 era

**DOI:** 10.3389/fsoc.2020.611885

**Published:** 2020-12-23

**Authors:** Roberto Lusardi, Stefano Tomelleri

**Affiliations:** ^1^Department of Human and Social Sciences, Università di Bergamo, Bergamo, Italy; ^2^Department of Human and Social Sciences, Università di Bergamo, Bergamo, Italy

**Keywords:** risk society, advanced modernity, neoliberalism, presentism, Bergamo, COVID- 19

## Abstract

This article arises from the urgent need to reflect on the current situation resulting from the dramatic consequences of a crisis which appears to be epochal and which, as sociologists, questions us at first hand. This is to understand the socio-cultural, economic and technological processes that triggered it and to attempt to imagine future scenarios. At the dawn of the third millennium, it seems as if the juggernaut of modernity, with its dream of unlimited progress and cargo of unconditional trust in instrumental rationality, has abruptly slowed down. The pandemic challenges contemporary society to develop a different *weltanschauung*, alternative to the performative and conformist idea of social planification supported by the neoliberal paradigm. It compels us to finally acquire the consciousness that the complexity of knowledge and global interdependency require collective awareness, political participation, and shared responsibility.

## Introduction: the Juggernaut Malfunction

This article arises from the urgent need to reflect on the current situation resulting from the dramatic consequences of a crisis that appears to be epochal. We propose a simple (and powerful) idea: to consider the COVID-19 pandemic as a cultural break point^1^. A watershed between the social configuration that we have known in the last 40 years and the one to come. It is now becoming evident that this pandemic has no equal in our recent past, in terms of the global extent of its spread, the restrictions on personal freedoms, the extent of the economic crisis, and the duration of the emergency situation (still uncertain). As sociologists, this situation questions us at first hand to understand the sociocultural, economic, and technological processes that triggered it in order to attempt to imagine future scenarios.

We would like to start from a photo which, in our opinion, captures the uniqueness and drama of the situation we are experiencing^2^: a long procession of Italian army trucks trawling through the streets of Bergamo, one of the Italian cities most affected by the COVID-19 pandemic, on the night of 18 March. Army vehicles were needed to take hundreds of coffins containing victims of the SARS-CoV-2 virus to other cities, as they could no longer be accommodated in the city cemetery facilities, which were already full. The photo had a striking impact on local citizens and on the rest of the country—it brought back the most tragic images of war, retreat, defeat, and death (Lusardi and Tomelleri, 2020).

At the dawn of the third millennium, it seems as if the *juggernaut of modernity* (Giddens, 1991), with its dream of unlimited progress and cargo of unconditional trust in instrumental rationality, has abruptly slowed down. The economic shock is challenging the sociological premises underlying the *fourth industrial revolution* being implemented or already operational in numerous production companies (Schwab, 2017). The sociological premises of this development model are the ones that have driven modernity up to now: the growth in the potential of instrumental rationality, which, according to Beck (1992), already contained many of the risks that we are faced with today; the almost unlimited trust in expert knowledge and in the transformative capacity attributed to technical and scientific progress (Giddens, 1991); and finally, the desire for strategic planning and control of natural phenomena and social processes, which was to ensure the stability of the system (Taleb, 2007).

The interdependence of scientific knowledge, technological application, and economic planning has consolidated over time, and they have mutually fueled one another. Likewise, trust in expert systems has steadily grown as scientific knowledge and technological innovation have provided new and powerful technical tools for manipulating nature to the point that, as Giddens affirmed, the juggernaut has become “a runaway engine of enormous power which, collectively as human beings, we can drive to some extent but which also threatens to rush out of our control and which could rend itself asunder” (1991: 139).

Beck and Giddens clearly state that thought on advanced modernity, driven by the extraordinary economic and social progress made since World War II, has overlooked the *latent collateral effects* or *undesired consequences* of industrialization and of the scientific, technological, and socio-economic infrastructure that supports it. This is not due to thoughtlessness or indifference. On the contrary, advanced modernity represents an unprecedented collective effort in economic and social planning. Faced with an environment undergoing rapid change due to technical and scientific progress, investments to guide its direction, control its developments, and, if possible, gain profit from it, become (Finn, 2017) priorities of scientific communities, industrial systems, and political-economic institutions. Big data, forecasting models, and algorithms are the most recent discoveries in a sector that has made the future (or rather, a certain idea of it, as we will see later) its field of work. The ability to record historical progressions and performance trends from which to inductively draw projections and inferences about the future has inhibited the capacity to conceive what is *unexpected* and what does not lie within the ranges of measurement of the most accredited instruments. This is what Taleb (2007) calls a *black swan*, or events of an exceptional rarity with devastating consequences, which are both unforeseen and surprising, in a much wider, reassuring, and predictable standard population of white swans.

The combined action of these three forces has, in fact, improved the lives of billions of people and allowed our species to make an unprecedented evolutionary leap, projecting the entire planet into the Anthropocene era in less than a century (Harari, 2015). It has also fueled the modern dream (or nightmare, for some) of world planning, for which *scientific determinism* is the epistemology of reference and *Laplace's demon* an effective metaphorical representation, to help understand why the unexpected has vanished from the horizon of thought on advanced modernity.

## The Perfect Machine

*Laplace's demon* represents a statement of the principle on which the idea of *scientific determinism* is founded: whatever occurs in the universe has a cause that generates future effects, and the more we expand our knowledge and empirical base, the more precise our predictions will become (Nekrašas, 2016). The future would no longer be uncertain and, like the past and the present, it would follow a single predictable and narratable flow (Prigogine and Stengers, 1979). Although numerous scientific discoveries and theories (from Einstein's relativity to the complexity theory) have challenged the determinism inherent in this ordering principle in different times and ways, modernity has remained deeply imbued with it (Morin, 1973).

This viewpoint perceives history as unfolding in a linear progression, social life in terms of rational planning, and government in terms of time and space, reducing society to decipherable and predictable schemes that consistently simplify its complexity (Giddens, 1991).

Trust in rational and instrumental technoscience is, however, a double-edged sword. It is at least problematic to uncritically raise the level of social expectations of technical and scientific knowledge toward a utopia where every individual and collective weakness can be solved and every limit overcome, including mortality (Bauman, 2001). In society, people encounter risky situations and experience the unpredictability of their choices and the uncertainty of the environmental and social conditions in which they act (Beck, 1992). The development of society and the degree of interdependence established between the various social arenas (economy, family, education, politics, etc.) do not imply the progressive elimination of risk and unpredictability. While admitting that the progress of technological and scientific means can predict future events with a certain approximation, it will never be possible to predict the extent, duration, and instance of the occurrence of an event with exact precision, as the tradition of *scientific uncertainty* affirms, because human beings are integral, variable, and dependent part of the systems they observes (Prigogine and Stengers, 1979).

However, social life is organized and reorganized following an arrangement of ideas and values where technoscience is crucial—the driving force of our inevitable destiny. Trust in technoscience hides the weaknesses experienced in our life, in our interpersonal relationships, in relationships between nations, and in relationships with ecosystems within an ideology of power and efficiency. The desire to foresee and plan the future becomes an obsession that cannot tolerate the uncertainty of history, which nevertheless remains in social life. The COVID-19 pandemic is a dramatic reminder.

## The Perversion of Modernity

The obsession with predictability and planning underwent sharp acceleration with the emergence of the neoliberal paradigm in economics from the 1980s onwards. With the economic and social policies of Margaret Thatcher in the United Kingdom and Ronald Regan in the United States, control and planning operated by (and on behalf of) state institutions were progressively replaced by forecasting devices based on the instrumental and efficiency logics of the market, which, according to neoliberal philosophy, require maximum freedom of self-regulation in order to guarantee the increase in wealth and the well-being of individuals and nations (Harvey, 2005). History shows us that it has not gone exactly like this: inequalities, exploitation, poverty, and pollution have paradoxically increased in recent decades, in the period of greatest wealth on the planet (Stiglitz, 2019). However, these side effects have not undermined confidence in the capacity for self-regulation of an economic system which, in order to prosper, required the systematic dismantling of external (political and social) constraints on its operation. The *deregulation* so dear to neoliberals consists of the following: the lightening or, better still, global suppression of the economic statutory and regulatory apparatus in order to allow the market to organize itself according to that paradigm that Friedrich von Hayek, one of the fathers of neoliberal thinking, called *spontaneous* (social) *order* (Whyte, 2019). If society (and above all the market, its pivotal institution) is able to independently develop a virtuous balance between individual interests and collective well-being—a balance not corrupted by what Hayek called the *fatal presumption*, namely, the presumption of having all the information necessary to make decisions for other people—any limitation of this spontaneous process becomes an obstacle to the achievement of wealth and well-being. It is a paradigm critical of both the Keynesian model of support for national economies and the social-democratic policies that lead to the main public welfare systems (Magatti, 2012). Thus, neoliberalism has favored the liberalization of markets (particularly financial markets), the globalization of goods and people, and the diffusion of mass consumption (Bauman, 2001). Any attempt by political institutions to introduce regulatory measures was seen as a threat to private property, individual entrepreneurship, and freedom of trade.

The social planning of state institutions has thus been progressively replaced by a material technostructure, supported by technoscientific development and the engineering of production and organizational processes that have colonized many aspects of the lifeworld (Beck, 1992). The freedom to act within an almost unlimited and ethically neutral market is linked to sophisticated forecasting technologies: neural systems, big data, algorithms, operating standards, and technological devices that improve the performance of the material technostructure and redefine social expectations, lifestyles, and consumption habits (Orrell, 2008).

Since the 1990s, the extensive and ever-increasing use of forecasting technologies on a planetary scale has contributed to the spread of a specific narrative regarding time and space, focused on the eternal present or presentism (Fukuyama, 1992). This narrative has relegated to the background, if not removed (at least until the start of 2020), the unpleasant awareness that our actions on the planet always have consequences (largely latent, unintentional, and unpredictable), which become all the more significant the more the systemic interdependence between science, industrial production, and consumption is emphasized for achieving greater profitability or new markets.

## The Eternal Present

Presentism bestows an exorbitant privilege on the present time, in a temporal perspective compressed into the short period, for which tomorrow will, in any case, always be the same as the present. Without the prospect of real change or a different future, the future is sacrificed to the benefit of immediate urgency. Men and women remove all references to the past and multiply their connections within an indefinite space of networks and a continuous flow of relationships and interactions, both virtual and actual (Maffesoli, 2000; Salmon, 2000; Taguieff, 2000). This flow of endless relationships and interactions reflects an inability to project into the future, transforming the uncertainty and unpredictability of existence into an increasingly precarious condition of life, without prospects of real change. Change is incorporated into a movement without respite, where the lack of an ultimate purpose or a goal to achieve transforms the *nascent state* (Alberoni, 1984) into a *permanent nascent state*. The precarization of life, therefore, does not only concern the process of changing employment contracts or working hours (Fourcade, 1992; Beck, 1998). Above all, precarisation implies frequent rearrangement of life stories, and consequent cultural reconfiguration, which makes all existing order unstable and precarious (Castel, 1995) and does not always contribute to regaining the deep meaning of one's own existential condition, which is increasingly fragmented and sectoral (Beck, 1998).

The desire to control the future tends to become an obsession with foreseeability that cannot bear the uncertainty of history. As Beck states, “the temporal horizons of perception narrow more and more, until finally in the limiting case history shrinks to the (eternal) present” (1992:135). Social time is flattened into the *eternal present*, and space is compressed due to the increasing speed required by the technostructure. The obsession with prediction is combined, as pointed out by Salmon (2000), with frenzy and speed. The contraction of time in the present is connected with its acceleration. The neoliberal economy, especially in finance, sees a competitive advantage in being the fastest. This is seen in the evolution of technology, which is constantly upgraded and enhanced, and in the development of economic and financial performance indicators, with an increasing focus on the present moment (from the quarterly results of companies, aimed at concentrating the effects of business plans, to stock market indexes that summarize real-time fluctuations in the financial markets) (Knorr and Preda, 2006). Continuous mobility, innovation, and transformation become moral imperatives, not with the aim of significant change in neoliberal society, but to devise ever-new adaptive strategies and increase profit. Events, like everything in life that is sudden and unexpected, are transformed into foreseeable and expected commercial products (often with long queues at the doors of the most famous brand stores). The surprises that are part of our experiences as men and women and, for better or worse, characterize what we define as living, are reduced to publicity stunts, repeated on each new occasion in accordance with the “eternal return” of presentism (Tomelleri, 2015).

Yet life, in its extremes of birth and death, remains an unexpected and unpredictable event. It is no coincidence that we can have no memory of these two extremes (Blumenberg, 1989). The unexpected and the unforeseen are the eruption of history into preplanned life and our social models. They are the eruption of chance into human affairs, they affect governments, economic systems, working conditions, school and university, and all aspects of social life. They disrupt all predictions about the future, exposing their illusory and delusional nature. These can be almost imperceptible moments, such as the birth of a child in Bethlehem, or epochal cataclysms, such as the COVID-19 pandemic, but its echo will most likely extend into the coming centuries.

## Conclusions: History Restarts

At the beginning of 2020, the unexpected appeared in the eternal neoliberal present. The COVID-19 pandemic represents a flaw in our capacity to forecast and plan ahead, marking an unexpected and unforeseen discontinuity. *The social and economic side effects of the side effects* of the current development model, to use an eloquent expression of Beck (1992), are jeopardizing the overall integrity of the global neoliberal system. Social isolation and a serious slowdown in productivity and consumption for an indefinite period of time, combined with the inability to envision future possible scenarios, are the unexpected consequences of an event that was anything but unpredictable. Cross-species virus spillover phenomena from animals to humans have already occurred in the recent past, and leading figures in the scientific and industrial world warned of the probability of this happening again. The unexpected, the black swan, bursts into the present through social and economic side effects that undermine the basic foundations of neoliberal societies (the free market, globalization, and mass consumption), whose stability was the essential condition for modern planning. The lack of future scenarios and the inconsistency of forecasting devices clash with the attempts to resort to the usual practices to govern the malfunction of the progress vector. The reiterated patterns of thought and behavior now prove to be ineffective for the paralysis of the socio-economic infrastructure. We are witnessing, to borrow a metaphor from Mills (1959), the crisis of the *cheerful robot* because we are being forced to face the possibility of the improbable, however tragic and dramatic this may be for the human species.

Famines, plagues, and wars have cyclically affected our ancestors, in all eras and geographical contexts, altering the course of history, changing societies, leading to the invention or dissemination of new technologies, and transforming the economy (Scheidel, 2018), and they have brought us to where we are today. The current situation reminds us dramatically that we are not so different from our ancestors. Perhaps we had forgotten about them because the second half of the twentieth century gave us an extraordinary period of wealth and freedom in which it seemed that these “enemies” of humanity had been defeated forever. By fostering the illusion of the human being as owner of its own sterilized and uncontaminated living environment, the neoliberal social model promised the impossible: to live the illusion of eternal youth in an indefinite present, where poverty, suffering, and pollution are just exotic filming locations for new broadcasting productions.

The image of the Italian army trucks transporting the bodies of the inhabitants of Bergamo who died during the acute phase of the epidemic collides with all these. It hits the juggernaut of modernity and the impact is disturbing, shocking, and devastating. The pandemic challenges us to develop a different *weltanschauung*, alternative to the performative and conformist idea of society supported by the neoliberal paradigm. It compels us to finally acquire an understanding that the complexity of knowledge and global interdependency requires collective awareness, political participation, and shared responsibility, as thinkers such as Morin (1973) had already suggested at the end of the last century. Some of these directions of change can already be glimpsed: extensive use of digital technologies is changing the way people work, study, and socialize; restrictions on global trade are relaunching national production chains (especially in agriculture and in the manufacture of essential goods); the fear of importing infection from abroad and rekindling new outbreaks will oblige us to review the international mobility of people and things; social relations will continue to be cautious for a long time, and, collective events (e.g., university exams, public competitions, soccer matches, concerts, and cinema) will change their form over the months and years to come.

Finally, the current situation, for the first time in our recent history, seriously challenges the category of the unexpected, so unsettling to those totally projected within the framework of the eternal present. The widespread confidence in the lasting stability of “today” made society insensitive to unforeseen occurrences, the impact of which is amplified by the inability of the system to detect (and accept) their magnitude. This phenomenon is complicated by collective resistance toward questioning the basic premises of contemporary society, already considered as consolidated and reliable. This highlights the paradox of modern rationalization: the more social organizations invest time, energy, and resources into planning, the more they trust what they do. And the more they find themselves unprepared when they have to face unexpected contingencies, which had not been taken into consideration, or which simply could not have been foreseen. Overcoming the probabilistic logic, which has contributed to erasing the side effects of industrialization during advanced modernity from our minds (but not from the planet), is an important preliminary step for collectively assuming the responsibility for a narrative and practice that acknowledges the precarious nature of human life, in an environment which encompasses us and transcends us.

We are convinced that we have just begun to glimpse the changes that the pandemic will bring to world societies. The hypothesis discussed in this article is intended as the starting point to stimulate further theoretical reflections or empirical research that explore some of the multiple implications of the pandemic in contemporary society. It will be essential to reconcile empirical observation with a perspective look to grasp the complexity and uniqueness of the historical period that we are experiencing. Further lines of research may concern both the sociological, epistemological and philosophical assumptions of the sociocultural change currently underway (e.g., elites and power in future democratic societies) and the socioeconomic phenomenologies that are growing to counter the spread of the virus and relaunch social life (e.g., the role of the third sector in supporting vulnerable people during lockdown).

## Author Contributions

All authors contributed to the article and approved the submitted version.

## Conflict of Interest

The authors declare that the research was conducted in the absence of any commercial or financial relationships that could be construed as a potential conflict of interest.

## Footnotes

^1^Note on method: The choice to point out the multiple theoretical, cultural and social implications of the pandemic, without scrutinizing every specific issue, stems from the need to provide a broad perspective of the phenomenon through an exercise of *sociological imagination*. This does not mean we disregard analytical rigor but has guided us toward a narrative style in which the theoretical discussion is part of a narration that proposes a global vision of the phenomenon, starting from our personal and professional observatory, to stimulate the “vivid awareness of the relationship between experience and the wider society” that characterizes sociologists' work (Mills, 1959: 6).

^2^The picture is available online at: https://www.europeansociologist.org/issue-45-pandemic-impossibilities-vol-1/na-reports-%E2%80%93-bergamo-march-2020-heart-italian-outbreak
